# Perilymphatic Fistula: A Review of Classification, Etiology, Diagnosis, and Treatment

**DOI:** 10.3389/fneur.2020.01046

**Published:** 2020-09-15

**Authors:** Brooke Sarna, Mehdi Abouzari, Catherine Merna, Shahrnaz Jamshidi, Tina Saber, Hamid R. Djalilian

**Affiliations:** ^1^Department of Otolaryngology–Head and Neck Surgery, University of California, Irvine, CA, United States; ^2^Department of Biomedical Engineering, University of California, Irvine, CA, United States

**Keywords:** perilymphatic fistula, perilymph fistula, dizziness, vertigo, tinnitus, association, blood patch

## Abstract

A perilymphatic fistula (PLF) is an abnormal communication between the perilymph-filled inner ear and the middle ear cavity, mastoid, or intracranial cavity. A PLF most commonly forms when the integrity of the oval or round window is compromised, and it may be trauma-induced or may occur with no known cause (idiopathic). Controversy regarding the diagnosis of idiopathic PLF has persisted for decades, and the presenting symptoms may be vague. However, potential exists for this condition to be one of the few etiologies of dizziness, tinnitus, and hearing loss that can be treated surgically. The aim of this review is to provide an update on classification, diagnosis, and treatment of PLF. Particular attention will be paid to idiopathic PLF and conditions that may have a similar presentation, with subsequent information on how best to distinguish them. Novel diagnostic criteria for PLF and management strategy for PLF and PLF-like symptoms is presented.

## Introduction

A perilymphatic fistula (PLF) is an abnormal communication between the perilymph-filled inner ear and outside the inner ear that can allow perilymph to leak from the cochlea or vestibule, most commonly through the round or oval window. PLF commonly causes cochlear and vestibular symptoms. Connections between vestibular symptoms and compromise of the structural integrity of the inner ear have been drawn as early as 1909 (1); however, vague symptoms, lack of a clear diagnostic test, and changes in the description and definition of a PLF have made even the existence of the condition a controversial subject for decades. In his work titled *Perilymph Fistula: Fifty Years of Controversy*, Hornibrook provides a detailed examination of the history of PLF and the sources of controversy surrounding the condition, including associated symptoms and terminology (2).

PLFs are ultimately a rare condition: it is estimated that PLFs has an incidence of 1.5/100,000 of adults, which is similar to that of vestibular schwannoma (3). In children, PLFs caused by congenital anomalies may be a more prominent cause of audiovestibular symptoms and have been thought to occur in up to 6% of children with idiopathic sensorineural hearing loss (4). Difficulties in defining and diagnosing PLFs has led to a dearth of more robust epidemiological information. Part of this problem has been that most methods used to identify PLFs lacked the sensitivity and specificity to provide consistent diagnoses (5). However, improvements in imaging techniques and emerging technology in the form of biomarkers have shown promise as tools to help define and diagnose PLF (6).

Despite their rarity, PLFs hold importance as one of the few potential causes of hearing loss and vestibular disturbance that can be treated surgically. In this review, we will present a compilation of current information on etiology and diagnosis of PLF as well as an update on new and developing treatment techniques.

## Etiology

PLFs can broadly be divided into two categories: those with an identifiable cause and those without. At first, PLFs were observed in post-stapedectomy patients where perilymph would leak around a prosthesis placed into the oval window due to a failure of the seal around or under the prosthesis (7–9). Though techniques for stapes surgery have advanced, PLFs still occur as a complication in ~1% of stapedotomy procedures (10) and may be present in up to one-third of individuals requiring revision stapedectomies (11).

Shortly after the identification of surgery as a cause of PLFs, Fee observed that PLFs may be present even with no history of prior otologic procedure and attributed their cause to head trauma (12). Potential causes include barotrauma, temporal bone fractures, and penetrating trauma (13–15). In the 1970s, Goodhill discerned implosive (originating from Valsalva force-induced increased pressure in the middle ear) from explosive forces [originating from increased cerebrospinal fluid (CSF) pressure] as causes of inner ear injury (16). In a study by Hidaka et al. that reviewed 51 causes of traumatic PLF in Japan, an estimated 40% were due to blunt head trauma, 35% due to penetrating injury, 5.8% due to barotrauma, and the remainder were iatrogenic (17). Interestingly, these numbers may vary by country, as the use of ear picks and Q-tips is generally higher in Japan (18).

There remained, however, cases in which individuals were found to have PLF symptoms with no history of either surgery or trauma. The exact amount of these “spontaneous” or “idiopathic” cases of PLF varies, but the number may be significant, ranging from 24 to 51% (19–21). Although these cases of PLF were historically called “spontaneous,” it is more accurate to refer to them as “idiopathic,” as the word “spontaneous” can have small but important differences in its definition that can affect how PLFs are classified (22). Occasionally these cases were still preceded by a specific event, such as sneezing, straining, nose blowing, laughing, or even bending over, prompting controversy over what constitutes an idiopathic PLF (22). Currently, there are no universally accepted formal diagnostic criteria for the diagnosis of PLF; however, in an effort to combat inexactness in the use of “spontaneous” PLF, researchers in Japan have created a modern classification system that divides PLF by cause into four groups (Table 1), similar to systems used in the past (16, 24). In this system, PLFs with antecedent events fall into categories 1, 2, and 3, while PLFs with no identifiable antecedent event fall into category 4 and are labeled idiopathic. Using this system, about 38.6% of cases in the study fell into category 4 (23). In contrast to Goodhill's classification (implosive vs. explosive) (16), the classification in Table 1 is simple and easy-to-use in clinical practice. In some cases of sudden deafness/dizziness following nose blowing, the route of inner ear injury cannot be discerned. In this scenario, nose blowing may increase either middle ear pressure via Eustachian tube (implosive) or intracranial pressure by straining (explosive); however, these types of mistakes cannot be made using the classification in Table 1.

**Table 1:** Categorization of perilymphatic fistula according to etiology based on a nationwide study by Matsuda et al. (23).

Category 1	Linked to trauma, middle and inner ear diseases, middle and/or inner ear surgeries
Category 2	Linked to barotrauma caused by antecedent events of external origin (such as flying or diving)
Category 3	Linked to barotrauma caused by antecedent events of internal origin (such as straining, sneezing, or coughing)
Category 4	Has no apparent antecedent event

The question of what may be provoking idiopathic PLF formation remains. In some cases, congenital malformations and microfissure formation may be a contributing factor (25). Microfissures can develop in multiple areas in the temporal bone, but those that develop between the round window niche and the posterior canal ampulla and around the oval window are theorized as an etiology for PLFs (26–29). Microfissures can be a normal finding (29); however, defective remodeling or anatomical variation in fissure location may distinguish fissures that contribute to PLF and those that are asymptomatic. In a similar manner, perilymph can leak through the fissula ante fenestrum as well. In normal development, the fissula ante fenestrum is a bony cleft present in all individuals that remodels and fills with cartilage and mesenchymal tissue. If this remodeling is altered, it may result in a patent cleft through which perilymph can leak (30). Elevations in intracranial pressure can also increase perilymphatic fluid pressure and cause or exacerbate fistulas (31). In many cases, patients may simply not recall a specific event preceding their symptoms. A more detailed discussion of potential factors is included at the end of this review.

## Diagnosis

Diagnosing PLFs has been a difficult task ever since their discovery over a century ago. Generally, they cause acute onset of audiological symptoms, vestibular symptoms, or both. This can include unilateral sudden hearing loss, tinnitus, vertigo, aural fullness, and disequilibrium (19–21). Commonly, patients present with both audiologic and vestibular symptoms, though they can be variable, and aural fullness in particular may be sensitive for PLF (6). There may be a history of head trauma, penetrating ear trauma, barotrauma, or prior otologic surgery. The audiovestibular symptoms can be similar in presentation to conditions such as superior or posterior canal dehiscence, vestibular migraine, endolymphatic hydrops, Meniere's disease, eustachian tube dysfunction, mal de debarquement, and persistent postural-perceptual dizziness, all of which similarly lack precise diagnostic tools. An expanded discussion of third window syndromes and how to distinguish them from PLF is included below. Clinicians should maintain high suspicion for a PLF when individuals with non-specific audiovestibular symptoms do not respond to conventional medical treatments or vestibular rehabilitation and when there is a history of onset after trauma or an inciting event (32). We have proposed a set of diagnostic criteria for aid in the identification of definite and probable PLF (Table 2).

**Table 2:** Proposed diagnostic criteria for perilymphatic fistula (PLF).

Definite PLF
Fluctuating or non-fluctuating hearing loss, tinnitus, aural fullness, and/or vestibular symptoms immediately preceded by one of the following events #1-3, which fulfills Criteria A or B:
Barotrauma caused by external events (e.g., slap/suction to the ear, head trauma, blast, skydiving, underwater diving, or flying, etc.) Barotrauma caused by internal events (e.g., nose-blowing, sneezing, straining, or heavy lifting, etc.) Direct trauma to the inner ear (e.g., Q-tip injury, stapedotomy operation, temporal bone fracture, etc.) Laboratory testing for a perilymph biomarker with high sensitivity and specificity. Observation of perilymph leakage in the middle ear and resolution of symptoms after treatment with intratympanic blood patch or surgical plugging of leak.
**Possible PLF**
Fluctuating or non-fluctuating hearing loss, tinnitus, aural fullness, and/or vestibular symptoms without antecedent event such as #1-3 above, with third window abnormalities and lack of response to migraine lifestyle, dietary, and prophylaxis therapy, and with resolution of symptoms after treatment with intratympanic blood patch or surgical plugging of leak.

For decades, the gold standard for diagnosis of a PLF has been intra-operative visualization of perilymph leakage with subsequent improvement in symptoms after the leak has been repaired. However, this test is arguably subjective as no established criteria exist for what constitutes a perilymphatic leak on observation (33). The total amount of perilymph in one inner ear is only slightly larger than three drops of water (~150 μl) (2, 32), prompting questions as to whether liquid observed in the middle ear could represent perilymph, CSF, or even local anesthetic and transudates (34). In our intraoperative observation, because the stapes footplate is placed in a dependent position in the middle ear during surgery, a small amount of transudate from the middle ear mucosa can accumulate in the footplate and create the appearance of a PLF when one does not exist. This transudate can increase as a result of manipulation of the middle ear mucosa or from the heat of a microscope, laser, or endoscope.

**Figure 1:**
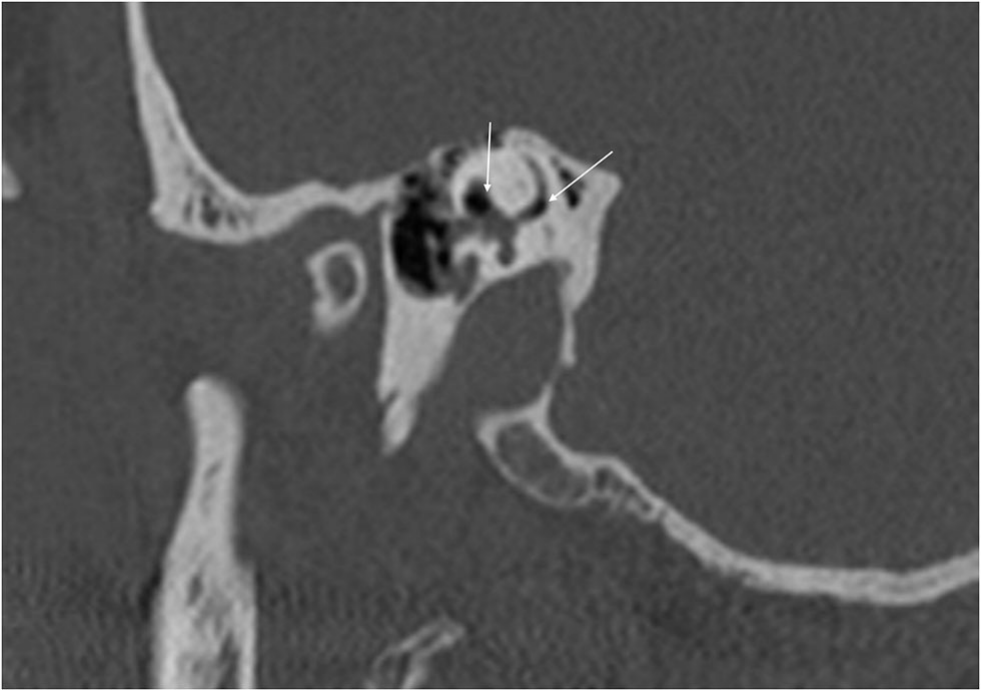
Sagittal CT of temporal bone demonstrating air in the vestibule and the crus communs (arrows) in a patient with perilymph fistula.

**Figure 2:**
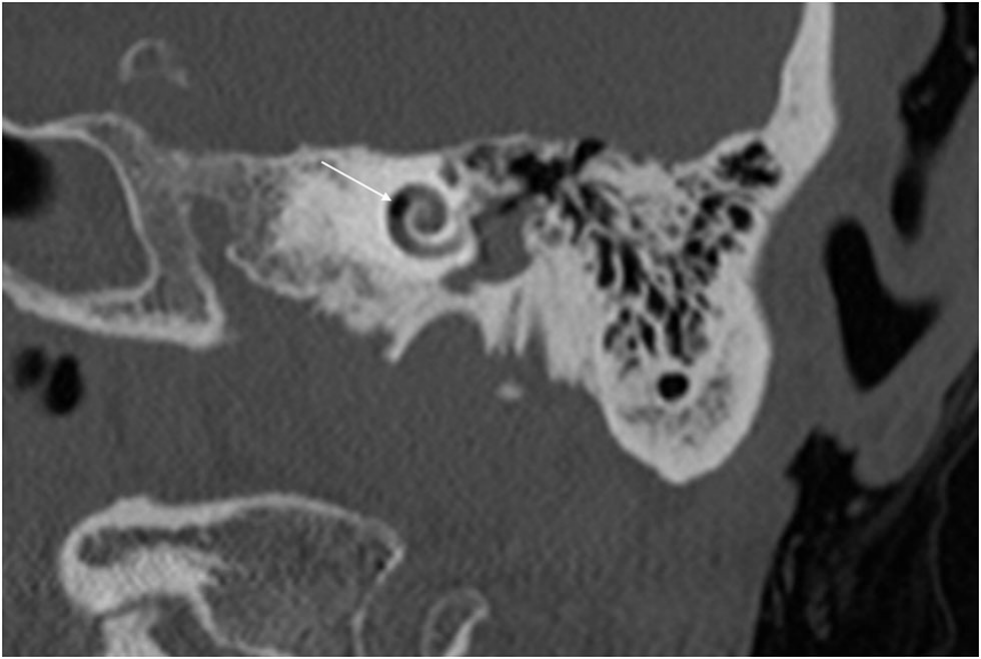
Coronal CT of temporal bone showing air in the second cochlear turn (arrow).

**Figure 3:**
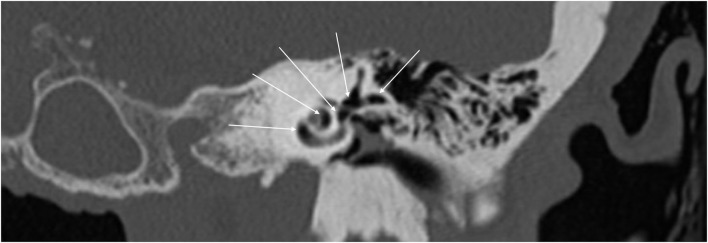
Coronal CT of temporal bone showing extensive air in the cochlea, superior canal, horizontal canal, and vestibule (arrows).

**Figure 4:**
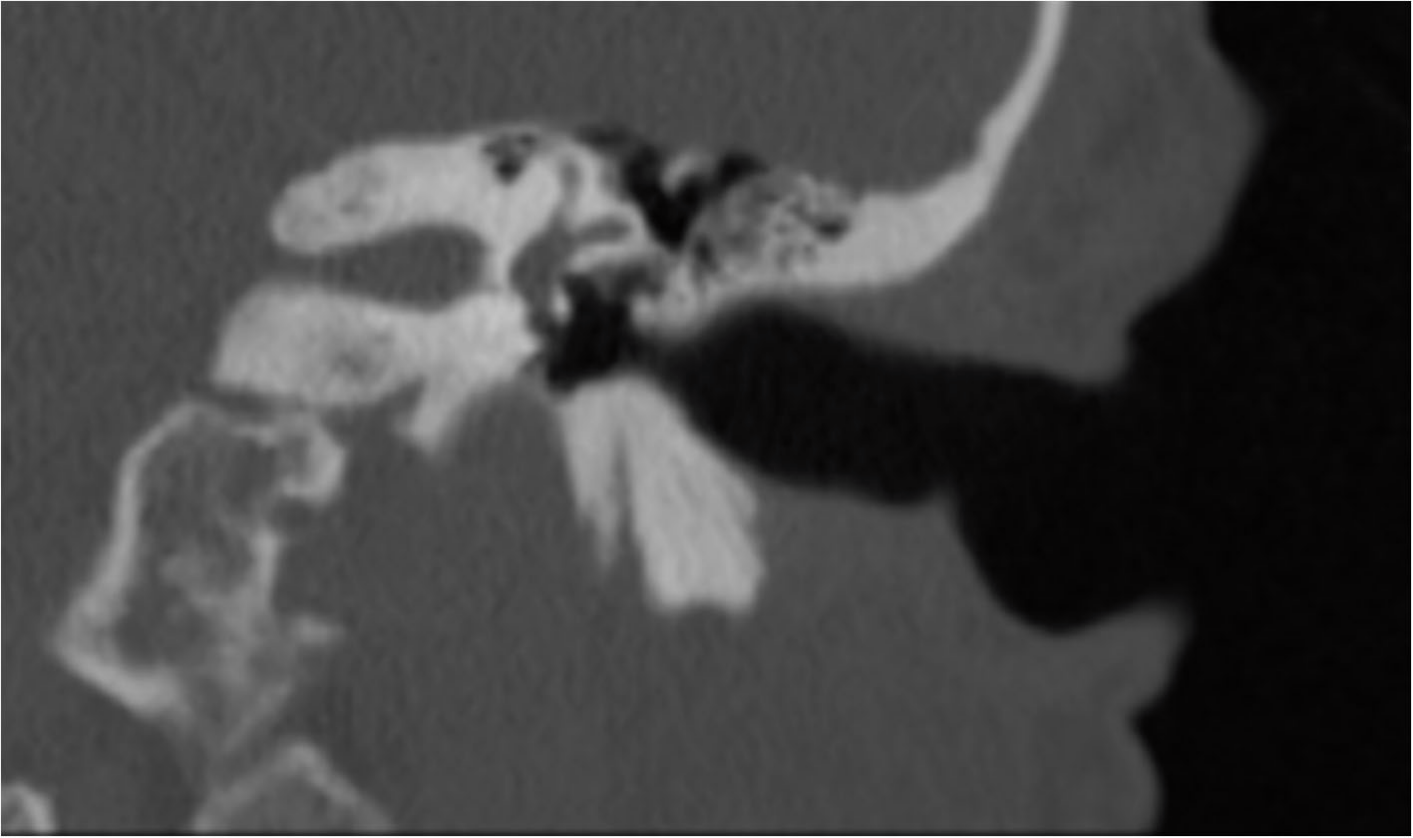
Coronal CT of temporal bone of the same patient in Figure 3 after perilymph fistula repair procedure. No air is seen in the inner ear.

**Figure 5:**
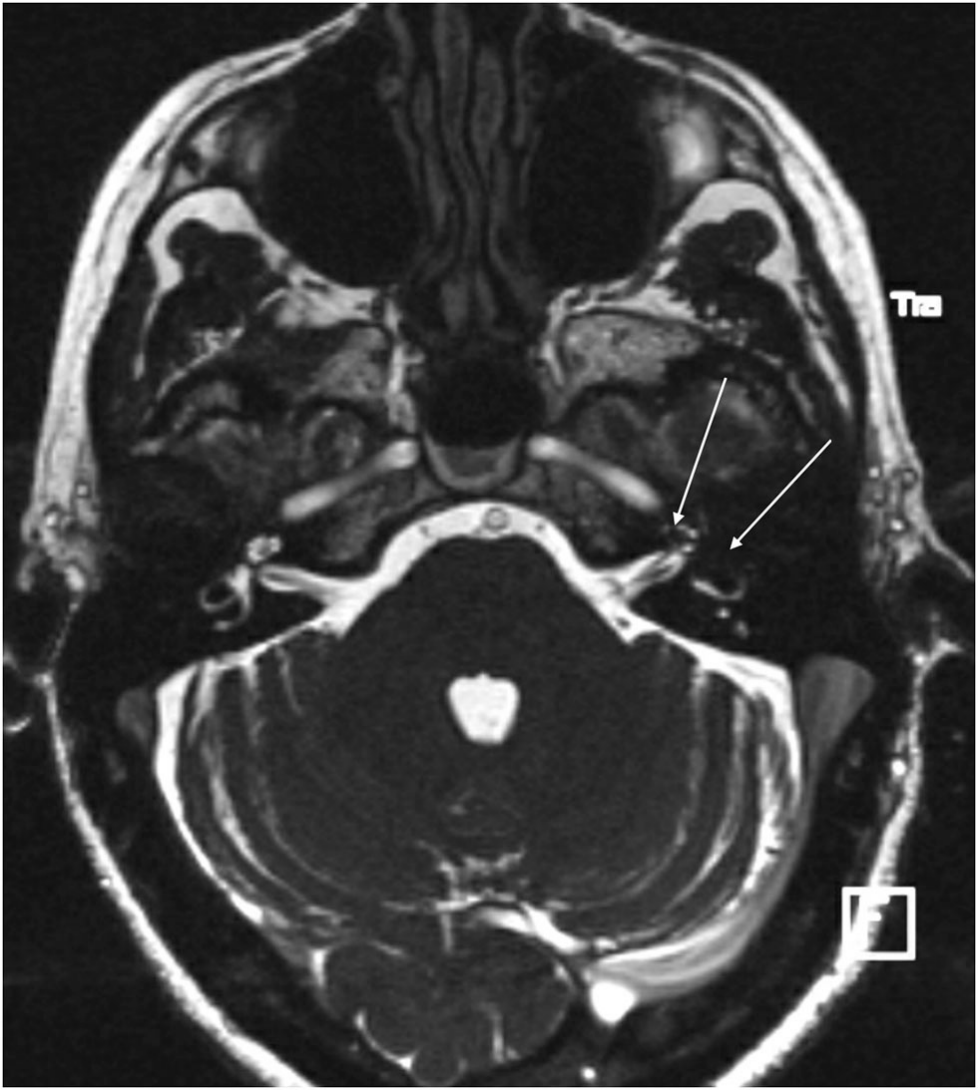
CISS sequence MRI of a patient with PLF showing significant air in the vestibule and the anterior crus of the horizontal canal as well as the second turn of the cochlea (arrows).

**Figure 6:**
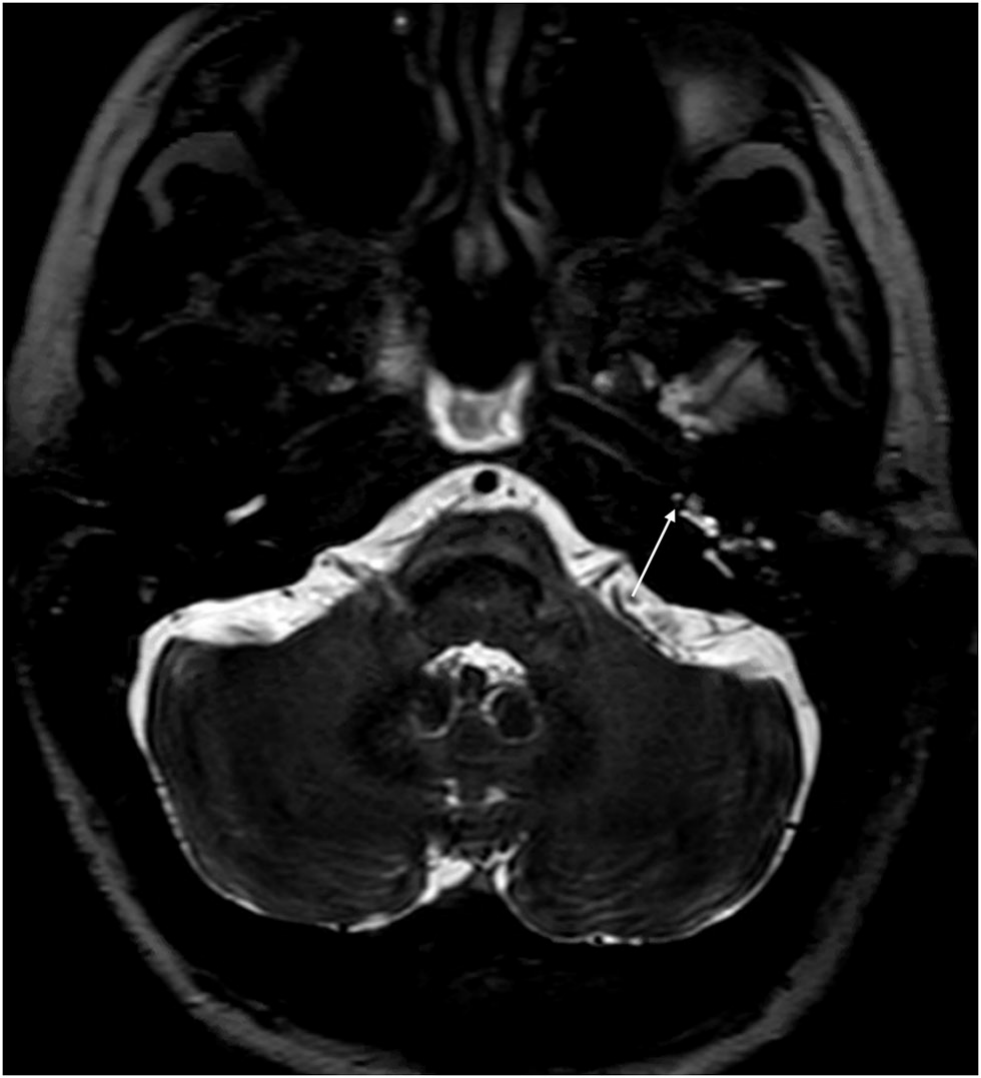
CISS sequence MRI of the same patient as Figure 5 1 day after blood patch procedure. There is a small amount of air in the distal basal turn of the cochlea (arrow).

**Figure 7:**
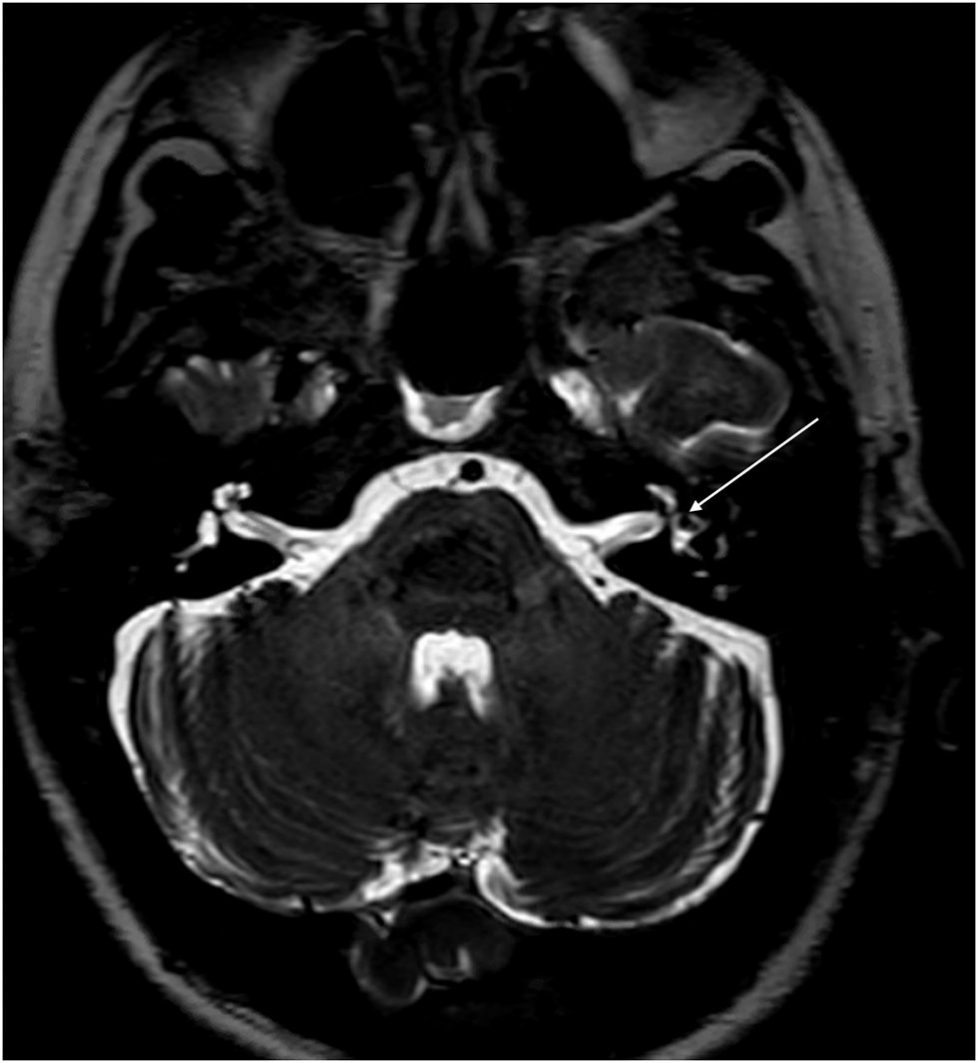
CISS sequence MRI of the same patient as Figure 6 at the level of the vestibule demonstrating improvement in the intravestibular air (arrows) compared to Figure 5.

**Figure 8:**
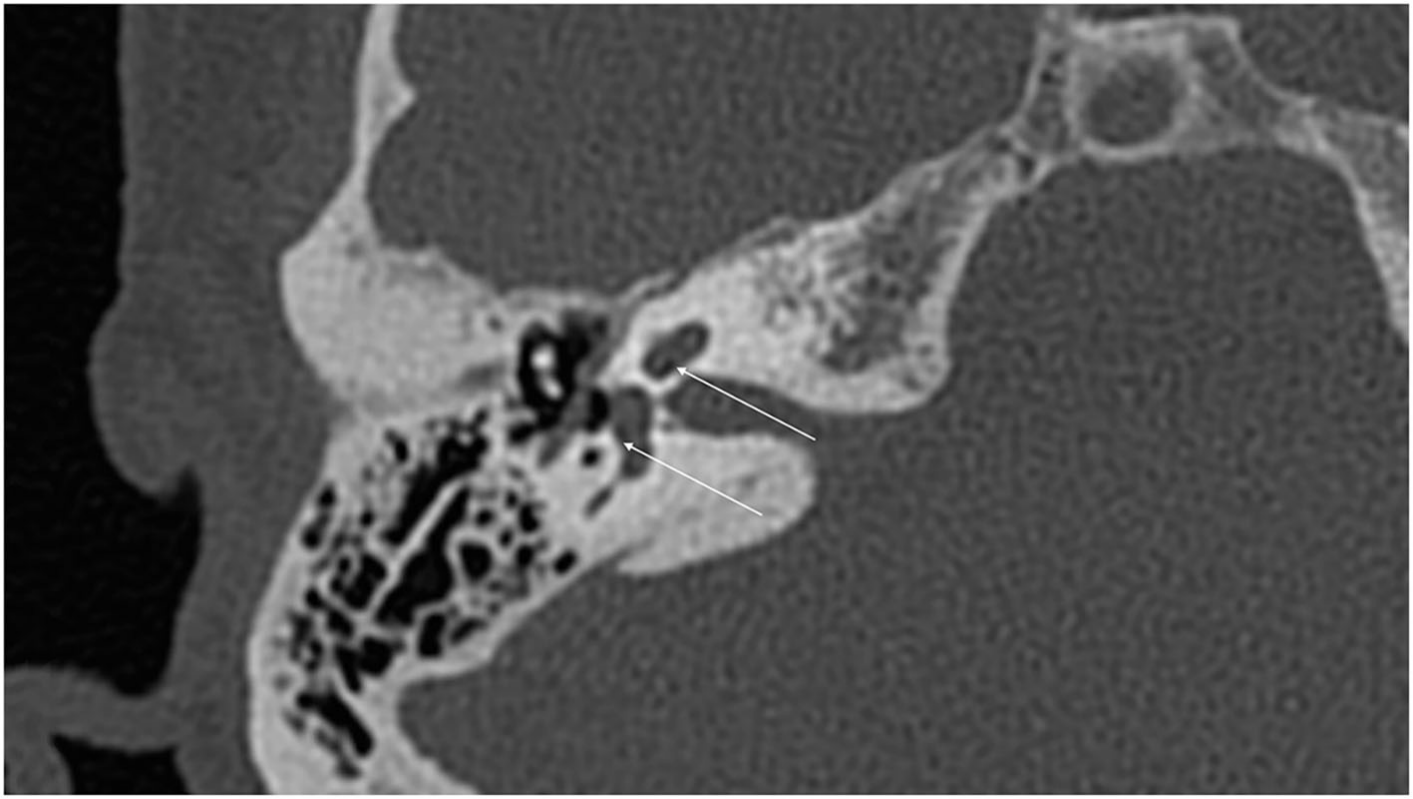
False positive hypodensities (arrows) seen in the cochlea on routine CT of temporal bone.

With the improvement in the resolution of computed tomography (CT) and magnetic resonance imaging (MRI), the need for exploratory procedures to identify PLFs in traumatic or post-surgical cases has declined. One of the earliest described radiological signs of a PLF is pneumolabyrinth, or air in the cochlea, vestibule, and/or semicircular canals (35). Small bubbles of air can be hard to visualize on typical CT scans, so high resolution scans including coronal or sagittal views may be useful in suspected cases (Figures 1–4) (13). Fluid in the round and oval window is another reliable sign of a PLF. A study by Venkatasamy et al. (36) evaluated the CT and MRI findings of 17 individuals with surgically confirmed PLFs and found that oval window PLFs most commonly presented with pneumolabyrinth and disorientation of the stapedial footplate, while round window PLFs most commonly presented with effusion of the round window niche. Generally, they found that high resolution CT scanning of the temporal bone has a sensitivity for detection of PLFs of over 80% when compared to intra-operative visualization of leak, and a combination of CT and MRI was reported to diagnose almost 100% of cases. We have found the axial and coronal CISS (constructive interference in steady state) (also called FIESTA (fast imaging employing steady-state acquisition) or MPR (magnetic resonance perfusion) sequence to be the most useful sequences (Figures 5–7). MRI may be particularly useful for identifying congenital abnormalities that may contribute to PLF formation and reduces the need for CT imaging in children. False negative cases may be due to scarring or intermittent or slow leakage of fluid, whereas false positive cases may be due to normal hypodensities seen in the cochlea (Figure 8), motion artifacts, or inflammation (36). Clinicians should be mindful of the context of sensitivity and specificity data for diagnosis of PLFs, as it is generally compared using visualization of leaks as a gold standard, which can be unreliable. Additionally, PLF can be intermittent in nature, increasing the amount of false negative cases. Imaging will generally be useful in acute post-traumatic or post-operative patients with larger leaks. In addition, CT is necessary in ruling out other causes of third window syndrome such as superior or posterior canal dehiscence, enlarged vestibular or cochlear aqueduct, and carotid or facial nerve-cochlea fistula, all of which can present similar to idiopathic PLF.

A variety of other testing methods have been used to help diagnose PLFs, including audiometry, cervical vestibular evoked myogenic potential (cVEMP), electrocochleography, and the fistula test, as part of videonystagmography. These methods have varying sensitivity for the diagnosis of PLF but generally may be helpful in localizing the affected side or in distinguishing nystagmus invoked by noise or pressure changes (6). The fistula sign is a clinical finding that has traditionally been used; a positive fistula sign is defined as nystagmus when negative pressure is applied to the external auditory canal. However, its sensitivity may vary from as little as 0% to as high as 77% (19, 32). The platform pressure test (PPT) is yet another specific tool lacks sensitivity that can be used to help diagnose PLFs (37).

New technologies are being continuously explored and developed that may shed light on precise diagnosis of PLFs. Virtual endoscopy is a method that recreates an intraoperative, endoscopic environment using three dimensional spiral CT scans. In a prospective study of 145 patients, Bozorg Grayeli et al. found that virtual endoscopy had a sensitivity and specificity of 75% for diagnosing PLFs when compared to intra-operative visualization or resolution of symptoms after surgery (38). It can be particularly useful for round window PLFs and for small PLFs <0.5 mm in size that are not visible on typical CT scans (39).

The use of biomarkers for the detection of perilymph fluid is similarly under investigation. Beta-2 transferrin and cochlin tomoprotein (CTP) have been targets of research as a potential way to confirm the leakage of perilymph within the middle ear. This test shows great promise and is continuously available as an investigator-initiated trial throughout Japan since first introduced by Ikezono et al. in 2009 (40). Recently in June 2020, the Japan Ministry of Health, Labor, and Welfare approved the CPT ELISA test which has qualities for medical diagnosis (personal communication). However, it still lacks regulatory approval for clinical use worldwide and appears to only be available by SRL Inc., Tokyo, Japan. This has limited its availability and adoption clinically. Beta-2 transferrin is a protein found in higher concentration in CSF, vitreous humor, and perilymph (41). Although some studies showed it may have been a promising marker to identify perilymph in the surgical environment (42, 43), other studies have raised concerns regarding ease of sample contamination with blood, blood plasma, CSF, and beta-1 transferrin (44). Unlike beta-2 transferrin, CTP is a protein found in perilymph but not in appreciable amounts in CSF (45). Western blot and ELISA testing of fluid and lavages from the middle ear for CTP shows promise as a reliable diagnostic tool for PLFs (23, 40, 46, 47). Currently, the test is limited by the presence of CTP in blood, which may represent a route for sample contamination; however, lavage techniques and centrifugation should dilute or remove any blood in the sample enough so as not to affect the final result of the CTP analysis (40).

## Treatment

Treatment of PLFs essentially falls into two categories: conservative or surgical. The management strategy chosen often depends on the etiology of the PLF and severity of symptoms. Generally, PLF with a known cause is a surgical disease; however, conservative therapy may be considered if no identifiable etiology for the PLF symptoms is known (idiopathic PLF) (32). Conservative therapy generally entails avoiding anything that can increase inner ear or intracranial pressure and potential use of intra-tympanic steroids in acute decompensation (6, 48). It is our belief that PLFs with a known cause should generally be treated surgically to avoid further degradation of hearing. PLFs without a known cause can be treated conservatively or surgically if conservative management fails. There is evidence, particularly in animal models, that some PLFs can heal on their own given adequate removal of factors that provoke high intracranial/intracochlear pressure such as straining (24, 49, 50). The precise characteristics of the PLFs that heal spontaneously have not yet been elucidated. Despite this, research appears to show that the more severe the inciting trauma, the lower the chance of spontaneous healing (51). It is difficult to know what percentage of patients benefit from conservative therapy alone as research in this area is lacking. We generally do not recommend conservative treatment in patients with known causes of the PLF, given the risk of progression to permanent hearing loss if surgical treatment is delayed (52, 53).

There is a spectrum of surgical treatment options ranging from in-office procedures to operations in a surgical theater, with the common goal of sealing the fistula. Typically, both the oval and round window are grafted using temporalis fascia or tragal perichondrium, regardless of which window contained the fistula. A variety of other materials have been used including fat grafts, areolar tissue, and Gelfoam (Pfizer, New York, NY) (19, 54). In patients operated on by other surgeons, we have seen significant conductive hearing loss when excessive fascia has been used around the oval window. We generally use Gelfoam around the oval window and fascia in the round window after creating a circumferential mucosal trauma with a needle or a defocused laser on low power, when we uncommonly have to perform surgery for these patients. We use fascia in the oval window only in cases of footplate fracture. In cases where an exploratory tympanotomy is used for diagnosis but no leak is visualized, historically up to 78% of clinicians reported that they would still graft the windows in consideration of an occult leak (55). Of note, this survey was conducted in 1990 and management strategies of current neurotologists may have changed.

**Figure 9:**
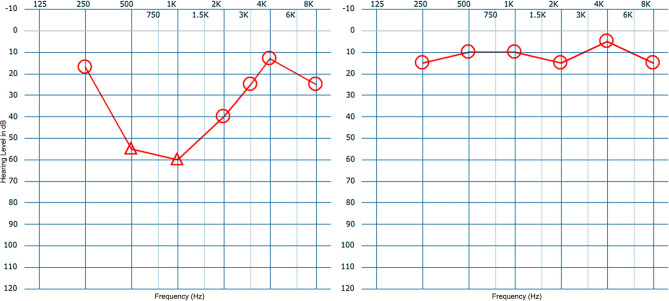
Audiograms of a patient presenting with sudden hearing loss after blowing her nose (left panel) showing improvement of hearing 1 week after a blood patch procedure (right panel).

Several years ago, a woman who was 12 weeks pregnant presented to us with acute vertigo and loss of hearing after she suffered trauma when a Q-tip was left in her ear. Examination showed trauma to the posterior superior quadrant of the tympanic membrane and a high frequency sensorineural hearing loss. The patient's case presented a dilemma: surgical treatment could place the fetus at risk, whereas conservative, non-surgical treatment could place her hearing at risk. The patient was offered the option of a blood patch procedure to potentially control the PLF. Under topical anesthesia, 0.5 cc of blood was injected into the middle ear and the patient was placed in a position so as to keep the oval window at its most dependent position for 30 min. The patient was given a suction to remove her saliva to prevent swallowing for the duration of the 30 min. The next day, the patient's vertigo had resolved, and her hearing had returned to normal. We have previously published a small report on the use of the blood patch procedure (56), and since our experience with those patients, we initially perform a blood patch procedure on all patients with suspected PLF. We generally do not perform VEMP testing prior to or after the blood patch procedure. All patients with a post-traumatic PLF have had resolution of their symptoms. This blood patch procedure is also used to rule out PLF in patients, particularly idiopathic PLF where a history of trauma is not present. In our experience, a lack of response to the blood patch procedure is likely suggestive of a lack of a PLF in the first place; however, it is important to be mindful of the fact that PLFs can resolve on their own with time as well as with conservative therapy and that surgical therapy may sometimes result in a negative response even in patients with a true PLF. Surgical therapy is only undertaken if there is a temporary response to the blood patch procedure with relapse of symptoms. The blood patch procedure is performed twice prior to performing a surgical procedure. Figure 9 demonstrate a typical improvement in hearing seen after a blood patch procedure. We theorize that initially blood covers the round and oval windows and seals them mechanically. After a few days, blood creates an inflammatory reaction that may facilitate granulation tissue formation and adhesion of adjacent tissues.

Surgery is generally effective at reducing or resolving patient symptoms, though vestibular symptoms tend to be improved more often than auditory symptoms. A range of 80–95% of patients experience improvement in vestibular symptoms, and a range of 20–49% experience improvement in hearing (19, 48, 57). The timing of surgery is a controversial subject—some authors recommend urgent corrective surgery within a few days of presentation (58), while others believe urgent surgery is not strictly necessary as improvements in hearing are small (48). Seltzer and McCabe reported that patients' hearing may benefit from surgery even after symptoms have been present for years (19), while other authors have found that prognosis may depend on timeliness of repair (53). The efficacy and timing of surgical repair depends on the particular etiology and location of the PLF. We generally recommend an in-office blood patch procedure upon presentation to the office or the emergency department.

## Discussion of Other Potential Causes and Associations of Idiopathic Perilymphatic Fistula

With potentially more than a third of PLFs being idiopathic in nature (23), it is important for the clinician to differentiate true PLFs from conditions that mimic PLF. It is likely that many cases of idiopathic PLF represent third window syndromes (59). The most common of the third window syndromes is semicircular canal dehiscence (SCD). The majority of canal dehiscence is seen in the superior canal (SSCD) (60), followed by the less common posterior canal dehiscence (PCD) (61). Horizontal canal dehiscence can be caused by chronic otitis media, fracture, neoplasm, or cholesteatoma. An idiopathic dehiscence of the horizontal canal, although rare, has also been described previously in the literature (62, 63). In SCD, thinning of the bone of the semicircular canals causes hearing loss, vertigo, and in some cases increased transmittance of bodily sounds (autophony) (60). The presence of autophony and the provocation of vertigo symptoms by sound or pressure are two features that tend to support a diagnosis of third window syndrome over a PLF.

In SSCD, there is no breakage of the membranes containing perilymph or endolymph in the inner ear, so no true membrane fistula is formed. Rather, the bone of the canal overlying the membrane is thin or dehiscent, creating a “third window” and resulting in symptoms. It was thought that thinning of the bone is likely congenital or developmental, as opposed to an acquired anomaly (64). However, newer evidence suggests that a higher body mass index (BMI) and obstructive sleep apnea (OSA) are more common in SSCD patients (65) This may be due to a higher intracranial pressure in patients with high BMI and OSA. Though the thin bone is present throughout life, symptoms do not appear until adulthood when trauma, erosion from the temporal lobe, and/or increased elasticity of the dura allows for pressure transference through the bone into the inner membranes (66). Only about 59% of patients with SSCD report a known inciting event (60)—the remaining 41% may present in a similar manner as an idiopathic PLF. SSCD can be distinguished from PLF by visualizing thinned bone over the superior canal on high resolution CT imaging using <0.7 mm slices, but it may be missed on conventional CT scans (67). Video head impulse testing may show decreased function of the affected canal (68). cVEMP testing will show lowered threshold values and increased amplitudes in both SSCD and PLF (69). Electrocochleography may also aid in diagnosis and will show an elevated summating potential (SP) to action potential (AP) ratio; however, this ratio will also be elevated in SSCD and Meniere's disease (70).

Other third window syndromes that can mimic canal dehiscence include carotid artery-cochlear dehiscence (CCD) and cochlea-facial nerve dehiscence (CFD). In CCD, there is thinning of the bone separating the carotid artery canal and the cochlea, most commonly between the basal turn of the cochlea and the petrous internal carotid artery (71). Though it can cause symptoms similar to both SCD and PLF, CCD usually presents with hearing loss and pulsatile tinnitus (72). MRI may not adequately visualize the internal carotid artery, so if suspicion for CCD is high, a high resolution CT scan should be obtained (73). Direct surgical repair of the fistula is not undertaken in these individuals due to proximity of the internal carotid artery (72).

CFD is a similar condition in which there is thinning of the bone between the cochlea and the labyrinthine segment of the facial nerve canal. It may also present with pulsatile tinnitus, fluctuations in or loss of hearing, and vertigo (74). CFD is rare—Fang et al. conducted a study on 1,020 temporal bone specimens and found complete dehiscence in only 0.59% (75). Of 401 temporal bones of patients presenting with a third window syndrome, Wackym et al. found 10.4% to have radiographically visible isolated CFD, with a further 7.8% having simultaneous CFD and another dehiscence (76). Like CCD, CFD is visible on high resolution CT imaging, but not all individuals with visible CFD on imaging will have associated symptoms. Direct surgical treatment of the dehiscence carries a risk of deafness and facial nerve paralysis—round window reinforcement is an alternative procedure that is effective at reducing vertigo and headache symptoms with fewer risks to important nerves (76).

There are third window syndromes which do not involve bony dehiscence, namely enlargement of the vestibular aqueduct (EVA) and enlargement of the cochlear aqueduct (ECA). EVA occurs as a result of a congenital malformation and often presents as mixed hearing loss in childhood (77). The third window effect may be one of many mechanisms via which EVA causes hearing loss (78). The conductive component of the hearing loss in EVA is likely due to the third window. Both MRI and high-resolution CT are sufficient for evaluating EVA (79); however, specific criteria for abnormal aqueduct width range from >1 to >2 mm (80, 81). ECA is a potentially related condition (82) with a similar mechanism of hearing loss. Unlike EVA, ECA is a rare condition that is steeped in some controversy regarding its existence and contribution to symptoms (83). ECA can generally be defined as a diameter >1 mm in the otic capsule portion and can be evaluated with high resolution MRI and CT imaging.

Another similarly presenting group of conditions is Meniere's disease (MD) and migraine. MD is a syndrome defined by a constellation of episodic vertigo, fluctuating hearing loss, tinnitus, and aural fullness. Patients can experience symptoms anywhere for a few minutes to as long as a day (84). Generally, there is a return to baseline between episodes; however, patients may have permanent progressive hearing loss over time. The cause of MD is still unknown, but it has one defining pathological feature: endolymphatic hydrops (85, 86). MD and PLF have been found in close association (87, 88), and PLF-induced changes in perilymph flows may alter the fluid production and balance in the inner ear so as to result in endolymphatic hydrops in some cases (89). Therefore, endolymphatic hydrops may be seen in both MD and PLF, and many patients with a PLF may have an element of MD as well. On the other hand, endolymphatic hydrops by itself does not appear to be sufficient to cause the symptoms of MD (86), and instead a complex interplay of factors including migraine, vascular changes, and interruptions in homeostasis may play a role (90).

**Figure 10:**
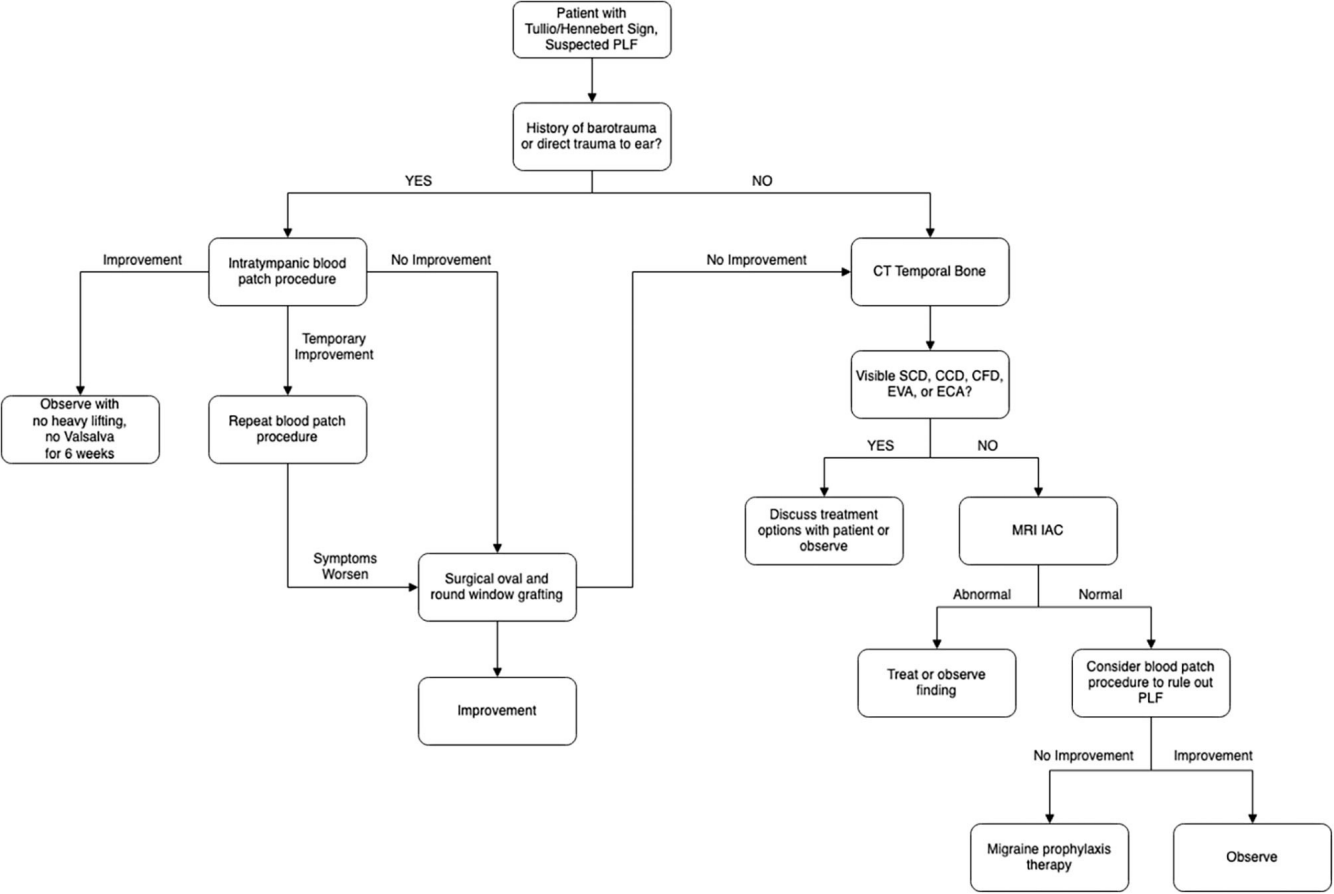
Algorithm for management of suspected perilymphatic fistula (PLF) based on the discussion provided in this review and the authors' experience. Patients with Tullio or Hennebert sign are entered into the algorithm. If the patients have barotrauma or direct trauma, they would be directed to the left side of the algorithm. If they do not have barotrauma or direct trauma, they would be then worked up/treated according to the right side of the algorithm. CT, computed tomography; MRI IAC, magnetic resonance imaging of internal auditory canal; SCD, semicircular canal dehiscence; CCD, carotid artery-cochlear dehiscence; CFD, carotid-facial nerve dehiscence; EVA, enlargement of vestibular aqueduct; ECA, enlargement of cochlear aqueduct.

Like MD, migraine can present with fluctuating hearing loss, and both conditions can have pressure change induced symptoms (91). There may be significant overlap between the two conditions, with up to 68% of individuals with MD experiencing migraine headache as well (92). Tympanostomy tubes, which equalize the pressure differential between the external and middle ear, may be a potential treatment option for pressure sensitive MD and migraine (93–96). It may be worth exploring other factors such as MD and migraine in seemingly idiopathic PLF patients who do not see significant benefit from window-sealing surgical treatment. A management strategy based on the experience of the authors for suspected PLF is summarized in Figure 10.

## Conclusion

Perilymphatic fistula is an enigmatic condition. Its diagnosis requires a thorough history to evaluate for a preceding event. For now, diagnosis and treatment choice continue to be based on an amalgam of clinical picture, vestibular, auditory, and imaging studies, and response to treatment, but advances in diagnostic criteria, high resolution imaging, and biomarker testing are paving the way for accurate pre-operative diagnosis in the near future. Similarly, surgical treatment techniques are progressing toward quick, in-office treatment for most cases. Though PLFs are rare, it is critical to remain vigilant of them as prompt treatment has the potential to save patients from debilitating vertigo and permanent hearing loss.

## Author Contributions

BS, MA, and HD contributed the conception and design of the study. BS, MA, CM, SJ, and TS performed review of the literature and collecting relevant information. BS, MA, and HD wrote the first draft of the manuscript. All authors contributed to the manuscript revision, read, and approved the submitted version.

## Conflict of Interest

The authors declare that the research was conducted in the absence of any commercial or financial relationships that could be construed as a potential conflict of interest.
